# Apical Ballooning Syndrome: A Complication of Dual Chamber Pacemaker Implantation

**Published:** 2009-07-01

**Authors:** Raed A. H Abu Sham'a, Elad Asher, David Luria, Michael Berger, Michael Glikson

**Affiliations:** The Heart Institute, Chaim Sheba Medical Center, Tel Hashomer, and the Sackler Faculty of Medicine, Tel Aviv University, Israel

**Keywords:** Pacemaker, Apical ballooning

## Abstract

Apical ballooning is a cardiac syndrome (Takotsubo Cardiomyopathy) described as a typical form of acute transient left ventricular dysfunction. While its onset has often been associated with emotionally or physically stressful situations, it has an overall favorable prognosis. We describe here a case of transient apical ballooning following permanent pacemaker implantation.

## Introduction

Apical ballooning syndrome (ABS) is a novel cardiac syndrome described recently as a typical form of acute transient left ventricular (LV) dysfunction associated with chest pain, ECG changes, and mild enzyme elevation without coronary occlusion [1]. While its onset has often been associated with emotionally or physically stressful situations, gradual recovery of LV dysfunction is the rule, and the overall prognosis is favorable. However, it could sometimes be associated with hemodynamic compromise and in rare cases may lead to serious complications and death [1,2]. We describe here a case of transient ABS following permanent pacemaker implantation.

## Case report

An 86 year old female, with no significant past medical history or cardiac symptoms, with the exception of a successful ablation of AVNRT 12 years ago, was admitted with recurrent syncope. She underwent a detailed evaluation, including an ECG and echocardiography, both of which were normal, with an estimated left ventricle ejection fraction (LVEF) of 60%. She was diagnosed by bedside monitoring to have intermittent complete AV block with ventricular escape rhythm of approximately 30 bpm.

**Figure 1:**
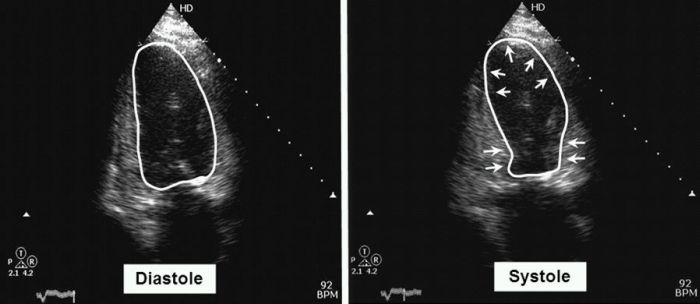
Echocardiogram clearly showing apical ballooning during systole with normal contraction of the basal segments (arrows)

**Figure 2:**
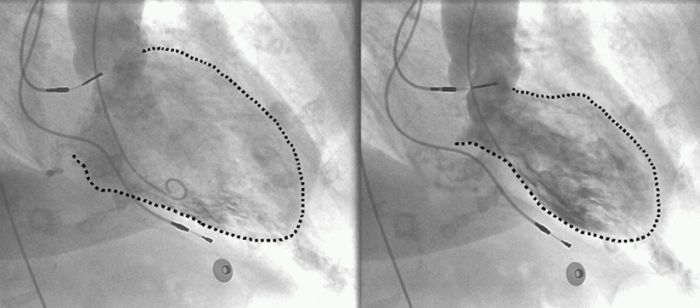
Left ventriculogram showing apical ballooning during systole with normal contraction of the basal segments (arrows)

She underwent implantation of a dual chamber pacemaker. The procedure was smooth with no apparent complications. On the night following implantation she started complaining of dyspnea and orthopnea which resolved spontaneously. She was discharged the following morning after pacemaker evaluation and chest X-ray, both of which were normal. Several hours later, however, she developed acute pulmonary edema. Emergency room ECG showed atrial synchronous ventricular pacing of approximately 80bpm, prolongation of the QTc interval up to 540 ms and non-specific ST-T changes. Echocardiography revealed normal left ventricle size, normal basal segment contraction with severe global left ventricular dysfunction and an estimated LVEF of 20%. The echocardiographic picture was typical of apical ballooning (Figure 1). Troponin I level was mildly elevated at 0.12 micg/L, (normal <0.07). The patient was managed conservatively; coronary angiography was performed after stabilization which showed normal coronary arteries, and left ventriculogram confirmed the diagnosis of ABS (Figure 2).

Over the next several days there was a dramatic improvement in her clinical condition, and a gradual recovery of LVEF on repeated echocardiograms. Repeated echocardiography after five days demonstrated an LVEF of 45%. She was discharged in stable condition and follow-up echocardiography demonstrated complete recovery of left ventricular function.

## Discussion

Apical ballooning syndrome was first described in Japan in early 1990s [3], where it was named Takotsubo Cardiomyopathy, due to the similarity between the dilated LV and an ancient Japanese fishing pot used to trap (tsubo) octopuses (tako). Earlier reports described such cases without applying the title of ABS [4,5]. Although the exact mechanism of this syndrome is unknown, it is thought to be due to excess catecholamine release, mainly norepinephrine, in stressful physical or psychological conditions and is thus named stress-induced cardiomyopathy  [5,6]. Alternatively, it could be due to transient coronary occlusion by spasm or ruptured plaque. However, the distribution of the affected areas does not follow a specific coronary artery anatomy, and coronary angiogram failed to show any evidence of plaque rupture or spasm even in patients with persistent ST-segment elevation  [7].

Pacemaker implantation could be associated with a number of acute complications related to the procedure, the patient and/or the device itself. However, we are aware of only three previous cases of ABS reported in the literature following pacemaker implantation [11,13]. In our case, there was no other cause for acute deterioration of LV function, and the diagnosis of apical ballooning syndrome was made and confirmed by applying the Mayo Clinic diagnostic criteria [14]. The classical course of gradual and complete resolution of the regional wall abnormalities and normalization of the LVEF at follow-up, helped to confirm the diagnosis. Notably, diagnosis may be more difficult in the absence of ECG changes that are masked by pacing, as was demonstrated in this case. We conclude that arrhythmia device implantation represents psychophysical stress that may be complicated by apical ballooning.
